# ^177^Lu radiolabeling and preclinical theranostic study of 1C1m-Fc: an anti-TEM-1 scFv-Fc fusion protein in soft tissue sarcoma

**DOI:** 10.1186/s13550-020-00685-3

**Published:** 2020-08-17

**Authors:** J. A. Delage, A. Faivre-Chauvet, J. K. Fierle, S. Gnesin, N. Schaefer, G. Coukos, S. M. Dunn, D. Viertl, J. O. Prior

**Affiliations:** 1 Radiopharmacy Unit, Department of Pharmacy, Lausanne University Hospital and University of Lausanne, Lausanne, Switzerland; 2 CRCINA, INSERM, CNRS, Université d’Angers, Université de Nantes, Nantes, France; 3 LAbCore, Ludwig Institute for Cancer Research, Lausanne University Hospital and University of Lausanne, Lausanne, Switzerland; 4 Institute of Radiation Physics, Lausanne University Hospital and University of Lausanne, Lausanne, Switzerland; 5 Department of Nuclear Medicine and Molecular Imaging, Lausanne University Hospital and University of Lausanne, Rue du Bugnon 46, CH-1011 Lausanne, Switzerland; 6 Ludwig Institute for Cancer Research and Department of Oncology, Lausanne University Hospital and University of Lausanne, Lausanne, Switzerland

**Keywords:** TEM-1, CD-248, Soft-tissue sarcoma, Theranostic, 1C1m-Fc, DOTA conjugation

## Abstract

**Purpose:**

TEM-1 (tumor endothelial marker-1) is a single-pass transmembrane cell surface glycoprotein expressed at high levels by tumor vasculature and malignant cells. We aimed to perform a preclinical investigation of a novel anti-TEM-1 scFv-Fc fusion antibody, 1C1m-Fc, which was radiolabeled with ^177^Lu for use in soft tissue sarcomas models.

**Methods:**

1C1m-Fc was first conjugated to p-SCN-Bn-DOTA using different excess molar ratios and labeled with ^177^Lu. To determine radiolabeled antibody immunoreactivity, Lindmo assays were performed.

The in vivo behavior of [177Lu]Lu-1C1m-Fc was characterized in mice bearing TEM-1 positive (SK-N-AS) and negative (HT-1080) tumors by biodistribution and single-photon emission SPECT/CT imaging studies. Estimated organ absorbed doses were obtained based on biodistribution results.

**Results:**

The DOTA conjugation and the labeling with ^177^Lu were successful with a radiochemical purity of up to 95%. Immunoreactivity after radiolabeling was 86% ± 4%. Biodistribution showed a specific uptake in TEM-1 positive tumor versus liver as critical non-specific healthy organ, and this specificity is correlated to the number of chelates per antibody. A 1.9-fold higher signal at 72 h was observed in SPECT/CT imaging in TEM-1 positive tumors versus control tumors.

**Conclusion:**

TEM-1 is a promising target that could allow a theranostic approach to soft-tissue sarcoma, and 1C1m-Fc appears to be a suitable targeting candidate. In this study, we observed the influence of the ratio DOTA/antibody on the biodistribution. The next step will be to investigate the best conjugation to achieve an optimal tumor-to-organ radioactivity ratio and to perform therapy in murine xenograft models as a prelude to future translation in patients.

## Background

The tumor endothelial marker 1 (TEM-1) also known as endosialin or CD248 is a type I single-pass transmembrane cell surface glycoprotein of 757 amino acids (80.9 kD) belonging to the C-lectin receptor superfamily. TEM-1 is composed of a signal leader peptide, five extracellular domains (including three EGF repeats), a mucin-like region, a transmembrane region, and a short cytoplasmic tail [1–3].

TEM-1 is expressed on mesenchymal lineage cells including pericytes and fibroblasts during tissue development, tumor neovascularization, and inflammation [4, 5].

Initially identified as the target antigen of an antibody (named FB5) raised in mice inoculated with human fetal fibroblasts, endosialin was found to be associated with the tumor vascular endothelium. TEM-1 expression has been localized to tumor vasculature, mainly in pericytes and stromal fibroblasts and in some cases to malignant cells [6, 7]. TEM-1 is implicated in tumor cell adhesion and migration, development, neoangiogenesis, and tumor progression [8, 9]. It has also been associated with tumor aggressiveness and poor patient prognosis [10, 11].

Studies with TEM-1 knockout mouse models were unaffected with regard to phenotype and wound healing responses, but showed an important reduction in tumor growth, invasiveness, and metastasis [12].

In human adults, TEM-1 expression is limited to endometrial stroma and occasionally fibroblasts, and has been shown to be upregulated in certain pathologies (including tumor progression and metastasis) [13].

TEM-1 has been described as an excellent therapeutic target since it is tumor specific, is associated with more aggressive tumor phenotypes, and its elimination (genetic or immune-mediated) leads to severe attenuation of tumor growth and metastasis without toxicity or any obvious phenotypic alterations [12, 14].

Soft-tissue sarcomas (STS) are a group of 50 different tumor entities arising from mesenchymal cells that exhibit great differences in terms of genetic alterations, pathogenesis, and clinical behavior [15]. Current treatment, besides surgery for local disease, comprises radiotherapy and chemotherapy.

Only a few patients may benefit from curative resection, however, and prognosis of metastasized or otherwise unresectable tumors is poor. For advanced stages, survival is less than 50% at 5 years [16]. The treatment for these advanced-disease patients is currently palliative.

Rouleau et al. analyzed 94 clinical sarcoma specimens and showed TEM-1 staining in 84% [17]. More recently, expression of TEM-1 was assessed in a group of 203 clinical sarcoma specimens and 96% of expression was reported [18]. Among many tumor types, sarcomas appear quite attractive for TEM-1 targeted therapy due to simultaneous expression of TEM-1 in the vasculature, stroma, and tumor cells [19].

Several endosialin targeting antibodies have already been developed for oncological applications. An anti-TEM-1, the MORAb-004 antibody, which is a humanized FB5 antibody has completed a phase-I clinical trial and is currently in phase II [20]. A few research groups have developed antibody-drug conjugates (ADCs) [21, 22] and a human antibody ScFv-Fc fragment has already been used for optical imaging and immunotoxin-based therapy [18, 23].

**Fig. 1:**
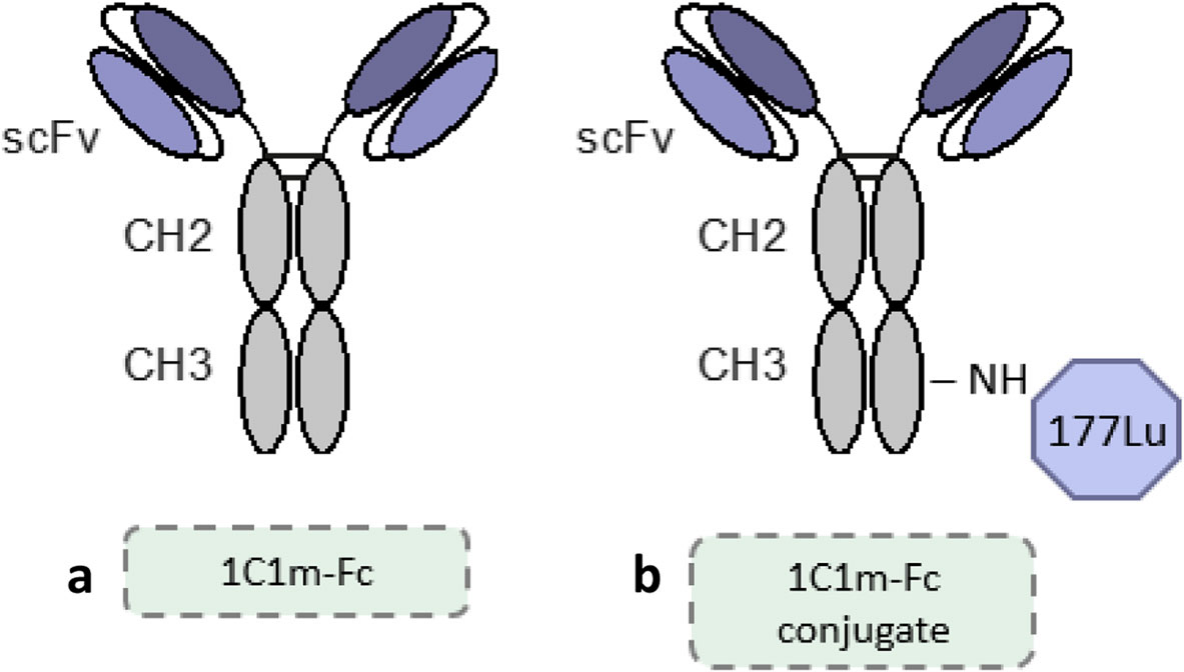
Schematic figure of 1C1m-Fc (**a**) and of 1C1m-Fc conjugate radiolabeled with ^177^Lu (**b**)

In this study, a fully human single-chain variable fragment (scFv) Fc-fusion, 1C1m-Fc, that cross-reacts with both mouse and human TEM-1 was used. 1C1m-Fc was conjugated to DOTA and labeled with ^177^Lu, a *γ* and *β*^–^ emitting radionuclide that can be used at low activity for diagnostic applications in single-photon emission computed tomography (SPECT), and high activity for therapeutic applications (Fig. 1). ^177^Lu is a favorable isotope for theranostic application with a half-life of 6.7 days, a maximal tissue penetration of 2 mm, and a low energy emission (*E*_*β*-max_ 0.49) that causes damage to neighboring healthy cells. ^177^Lu emits 2 photons at 113 KeV (6.4%) and 208 Kev (11%) which allows both imaging for monitoring and dosimetry of the same compound [24].

As anti-TEM-1 imaging could both detect tumors and monitor responses, and could help identify patients suitable for targeted therapy, we decided to perform in vitro and in vivo preclinical evaluations of this novel fusion protein antibody.

## Materials and methods

### Fusion protein antibody

1C1m-Fc, also named HS06 mut (molecular weight 106196.8 Da, molar extinction coefficient 162830 M^−1^ cm^−1^ at 280 nm) was isolated by phage display from a naïve human antibody phage display library at the LAbCore immunoglobulin discovery and engineering facility, Ludwig Institute for Cancer Research, Lausanne. The scFv was made bivalent by fusion to a human Fc domain (IgG1). The fusion protein was expressed and secreted from HEK293-6E cells, purified by Protein A affinity chromatography, and buffer exchanged into PBS. Affinity to human and murine TEM-1 was respectively 1 and 6 nM [25].

### Cell lines

The murine endothelial 2H-11 (TEM-1 positive), human neuroblastoma SK-N-AS (TEM-1 positive), and human fibrosarcoma HT-1080 (TEM-1 negative) cell lines were purchased from the American Type Culture Collection (ATCC, Manassas, VA, USA).

All cell lines were cultured in DMEM (Thermo Fisher Scientific, Waltham, MA, USA) supplemented with 10% fetal bovine serum (FBS, Thermo Fisher Scientific, Waltham, MA, USA) and 1% penicillin/streptomycin (Thermo Fisher Scientific, Waltham, MA, USA). Cells were incubated in a flask at 37 °C in a humidified atmosphere at 5% CO_2_.

### Conjugation

Absorbance at 280 nm of the fusion protein antibodies was measured using a spectrophotometer (NanoDrop Lite, Thermo Fisher Scientific, Waltham, MA, USA), and the molar concentrations were obtained from the absorbance and the respective molar extinction coefficients (M^−1^·cm^−1^).

1C1m-Fc was conjugated with increasing ratios from 10 to 40 equivalents of p-SCN-Bn-DOTA (Macrocyclics, Plano, TX, USA) using the following procedure: after conditioning a concentration of 5 mg/ml of 1C1m-Fc in carbonate buffer 0.2 M pH 9.0 by three ultrafiltrations on 50 kD membrane (Amicon Ultra, 0.5 mL, 50 kD, Merck, Darmstadt, Germany), a calculated volume of a solution of p-SCN-Bn-DOTA at 1 equivalent per μl in a mixture of 50 μl of dimethyl sulfoxide (DMSO) and 450 μl of carbonate was added to the buffered 1C1m-Fc solution. Mixtures were incubated for 1 h at 37 °C, and the conjugated antibodies were washed by four ultrafiltrations using PBS pH 7.4 before performing high-pressure liquid chromatography (HPLC) to assess integrity of the conjugates. Material was subsequently stored between 2–8 °C.

### Mass spectrometry analysis

The mass spectrometry (MS) analysis was performed using a Q Exactive™ HF Orbitrap with BioPharma option (Thermo Fisher Scientific, Waltham, MA, USA) operating in the high mass range. The mass spectrometry spectra were deconvoluted using the Protein Deconvolution Software (Thermo Fisher Scientific, Waltham, MA, USA). UPLC was also performed on the samples. The separation was done using the MAbPAC SEC-1 column, 5 μm, 300 Å, 4 × 150 mm (Thermo Fisher Scientific, Waltham, MA, USA), and ammonium acetate 50 mM pH 7.0 at 0.3 mL/min as mobile phase. By knowing the average mass of the antibody and the center of the conjugated antibody average mass distribution (broader peak in MS spectrum than the unconjugated form), an average number of chelators linked to the antibody was calculated.

### Radiolabeling

The radiolabeling was optimized with 500 pmoles of DOTA-conjugated 1C1m-Fc and 20 MBq of ^177^Lu (without carrier, EndoleucineBeta 40 GBq/ml, ITM, in aqueous 0.04 M HCl solution) in acetate buffer 0.4 M pH 5.6. After 1 h incubation time at 37 °C, the radiochemical purity was determined by instant thin layer chromatography (ITLC) and by HPLC.

### Purity and stability

The 1C1m-Fc candidate was tested for chemical purity by reducing and non-reducing SDS-PAGE using NuPAGE Bis-Tris gradient gels. The purity and the stability of the native and conjugated fusion protein antibody were also evaluated at 3, 6 months, and 1 year by HPLC. The profiles at the given timepoints were compared to the initial chromatogram.

The stability of [177Lu]Lu-1C1m-Fc in human serum was also assessed at 24 and 48 h by iTLC.

### HPLC

HPLC analyses were performed using an Ultimate 3000 SD System (Thermo Fisher Scientific, Waltham, MA, USA) coupled to a GabiStar detector (Raytest, Straubenhard, Germany). Compound were separated with a size exclusion column, XBridge protein BEH 200 Å SEC 3.5 μm, dimension 7.8 × 300 mm (Waters, Baden-Dättwil, Switzerland). Elution was performed using phosphate buffer pH 6.8 (1 mL/min) as mobile phase and was monitored via absorbance at 220/280 nm or *γ* detection.

### iTLC

iTLC analysis were performed using dried iTLC-SG Glass microfiber chromatography paper impregnated with silica gel (Agilent Technologies, Folsom, CA 95630).

Detection of the radioactivity were obtained on a miniGITA scanning device (Raytest, Straubenhard, Germany) using the Gina star software after manual integration of the peaks. In this system, the [177Lu]Lu-1C1m-Fc remain at Rf = 0 while the unbound ^177^Lu migrate to the solvent front.

### In vitro characterization

#### Flow cytometry

1C1m-Fc and its conjugates were tested for binding to TEM-1 using FACS analysis. Either human cell lines (SK-N-AS or HT-1080) or murine cell lines (2H-11) were distributed in a 96 well plate (100 μl at 0.5 × 10^6^ per mL). After spinning down, the wells were washed once with 100 μL of flow cytometry staining buffer (PBS containing 2% FBS) and the cells were incubated with this FACS buffer (10-30 min) to block any unspecific binding. 1C1m-Fc or its conjugates (from 0.2 μg/mL to 2 μg/mL) were then added and incubated at 4 °C for 45 min. After washing, 50 μL of the secondary antibody (anti-human Fc, Alexa Fluor 647, Thermo Fisher Scientific, Waltham, MA, USA) was added with incubation in the dark for 30 min at 4 °C. Cells were washed and re-suspended in FACS buffer before being analyzed using a BD LSR-II (BD Biosciences) flow cytometer. The secondary antibody and unstained cells were used as negative controls. Median fluorescence intensity (MFI) was studied for 1C1m-Fc and its conjugates.

#### Radio-immunoreactivity

The immunoreactive fraction was assessed using Lindmo assay [26]. A fixed concentration of radiolabeled 1C1m-Fc (0.07 μg/mL) was incubated with increasing numbers (0.25-8 x 10^6^) of SK-N-AS cells in PBS containing 0.5% BSA (PBS/BSA) for 3 h at 37 °C on a shaking platform. Non-specific binding was evaluated by the addition of an excess of native non-radiolabeled 1C1m-Fc (> 100-fold excess).

Unbound activity was washed away twice with PBS/BSA after centrifugation for 5 min at 300 g. The cell-bound activity was measured with a gamma counter (AMG Automatic Gamma Counter, Hidex, Turku, Finland).

All conditions were tested in triplicate. The binding curve was extrapolated to an infinite number of cells using nonlinear regression from the Graphpad Prism 8.0 software (GraphPad Software, San Diego, CA, USA).

### In vivo characterization

#### Murine xenograft model

All animal experiments were conducted in compliance with the cantonal authorization VD-2993 and the guidelines of the Institution.

Tumors expressing huTEM-1 were established by subcutaneous injection of 3 × 10^6^ SK-N-AS cells in mouse flank of 6–10-week-old female Balb/c nude mice (Charles River Laboratories, Wilmington, MA, USA). A negative control was also obtained with injection of 3 × 10^6^ HT-1080 cells (TEM-1 negative).

Tumors were allowed to grow to 5-10 mm (largest diameter) before initiating studies. For SPECT imaging, some mice were injected with both TEM-1 positive and negative tumors. In this case, and due to differences in tumor cell growth rate, injection of HT1080 cells (3 x 10^6^) was delayed by 10 days.

#### Saturation assay

To assess the non-specific targeting and to optimize the dose to inject for the biodistribution studies, a blocking experiment was performed. Mice bearing SK-N-AS tumors were injected in the lateral tail vein without anesthesia with 100 μl of a saline solution containing 2.5 μg of [177Lu]Lu-1C1m-Fc conjugated with 3 DOTA and an increasing amount of unlabeled native 1C1m-Fc (respectively 2.5, 50, 100, 200, and 500 μg).

In each group, three animals were euthanized by CO_2_ inhalation and exsanguinated at 24 h after injection of the radiolabeled product. Blood was collected, organs and tumors were removed, weighed, and counted with a gamma counter (AMG Automatic Gamma Counter, Hidex, Turku, Finland).

#### Biodistribution studies

[177Lu]Lu-1C1m-Fc conjugated with 3 DOTA was injected into the lateral tail vein of the mice without anesthesia and sterile filtration. Animals were divided into 2 groups, the average weight of animals was 20.18 ± 1.7 g. Group 1 received an injection of 200 μg of a non-specific unlabeled human immunoglobulin Kiovig™ (Shire, Switzerland GmbH) on the first day (D0) and a mixture of 2.5 μg (1 MBq) of 3 DOTA’s [177Lu]Lu-1C1m-Fc and 47.5 μg of unlabeled 1C1m-Fc the day after (D1). Group 2 received a mix of 2.5 μg (1 MBq) of 3 DOTA’s [177Lu]Lu-1C1m-Fc and 47.5 μg of unlabeled 1C1m-Fc at D1, without Kiovig™ injection at D0. The volumes for all the injection were 100 μl, sodium chloride was used to perform the dilution.

In each group, three animals per time point were euthanized by CO_2_ inhalation and exsanguinated at 4, 24, 48, 72 h, and 6 days after injection of the radiolabeled product. Blood was collected, organs and tumors were removed, weighed, and counted with a gamma counter (AMG Automatic Gamma Counter, Hidex, Turku, Finland).

A second experiment was performed with the same conditions as for group 2, but with [177Lu]Lu-1C1m-Fc conjugated with 6 DOTA.

Results were expressed as the percentage of injected activity (IA) per gram of tissue (%IA/g).

#### Animal imaging study

Three hours static images were acquired with a small-animal PET/SPECT/CT (Albira, Bruker Biospin Corporation, Woodbridge, CT, USA). Mice of two groups (with or without Kiovig™ at D0) were injected with 50 μg corresponding to 18.5 ± 1.8 MBq of [177Lu]Lu-1C1m-Fc via tail vein injection. Mice were anesthetized for the duration of the imaging sequence by inhalation of 1.5% isoflurane/O_2_ and placed on a heated bed. SPECT/CT images were acquired at 24, 48, and 72 h after injection of the radiolabeled antibody for mice with either TEM-1 positive tumors, TEM-1 negative tumors, or both. The acquisition parameters were for SPECT: 80 mm transversal field-of-view, with a single pinhole collimator and for CT: 400 μA intensity and 35 kV voltage. Six animals were imaged: four belonging to group 1 with Kiovig™ saturation (respectively, one with a TEM-1 negative tumor, one with a TEM-1 positive tumor, and two with both TEM-1 positive and negative tumors), and two belonging to group 2 without Kiovig™ saturation (one with a TEM-1 positive tumor and one bearing both TEM-1 positive and negative tumors). For two animals belonging to group 2, imaging at 72 h was performed and then the mice were sacrificed to allow a biodistribution study. The image reconstruction methods were for SPECT: ordered subset expectation-maximization algorithm, 2 iterations and with scatter correction, and for CT: filtered back-projection algorithm with de-ringing correction. The tumor volumes of interest (VOI) were obtained by manual segmentation on axial CT slices using the PMOD software (PMOD technologies, version 3.709, Zurich, Switzerland).

#### Mouse dosimetry

Estimated absorbed doses to organs were based on the biodistribution results on SK-N-AS bearing mice of group 2. Considered source organs for the biodistribution study were the liver, the kidneys, the lungs, the spleen, the heart, the stomach, the small intestine, the colon, the urinary bladder, and the total body. We obtained the reminder by subtraction of the signal measured in source organs from the total body. For each mouse at each time point, the activity in each source organ and the remainder was normalized by the total injected activity to obtain the normalized injected activity (nA). For each source organ at each time point, an average nA value was obtained ± SD.

We fitted the source organs normalized time-activity curves (nTACs) with bi-exponential functions using the kinetic module of OLINDA/EXM 2.1 (HERMES Medical Solution AB, Stockholm, Sweden). We derived time-integrated activity coefficients (TIACs) by analytical time-integration of fitted source organ nTACs obtained with the average nA, nA + SD and the nA–SD values, respectively.

A specific absorbed dose estimated was obtained for the uterus, this organ, in fact, exhibited an important specific tracer uptake, but was not among the source/target organs available in the murine model of the OLINDA/EXM 2.1 software.

In the liver, the stomach, the bladder, the uterus, and in the TEM-1 positive tumor, the radioactivity was still in the uptake phase 48 h post-injection. For these tissues, the TIAC was obtained by trapezoidal integration using the Matlab software (release 2017a, The MathWorks, Inc., Natick, Massachusetts, USA), between *t* = 0 and *t* = 6 days, whereas a mono-exponential analytical integration to infinity was calculated after the last measure (*t* > 6 days) considering the ^177^Lu physical decay constant.

Finally, the TIACs were entered into the OLINDA/EXM® 2.1 software kinetic module for organ absorbed dose estimates considering the 25 g murine model where the phantom source organ masses were adjusted to the average organ masses obtained from the mice population used in our experiment. In this process, the TIAC of the uterus was part of the remainder of the body.

A specific absorbed dose estimate for the uterus was obtained using the sphere model of OLINDA/EXM 2.1 where the average organ TIAC and the average organ mass were applied.

### Statistics

The data are expressed as mean ± SD. Significant differences between means were analyzed by an unpaired, 2-tailed Student *t* test with a correction for multiple comparison using the Holm-Sidak method (*α* = 0.05). Curve-fitting and statistical analyses were conducted using Prism 8.0 (GraphPad Software, San Diego, CA, USA).

## Results

### Conjugation, radiolabeling, and stability tests

SDS-page of native 1C1-m and of 1C1m-Fc conjugated with 3 and 6 DOTA was performed (Fig. 1, supplementary data).

**Fig. 2:**
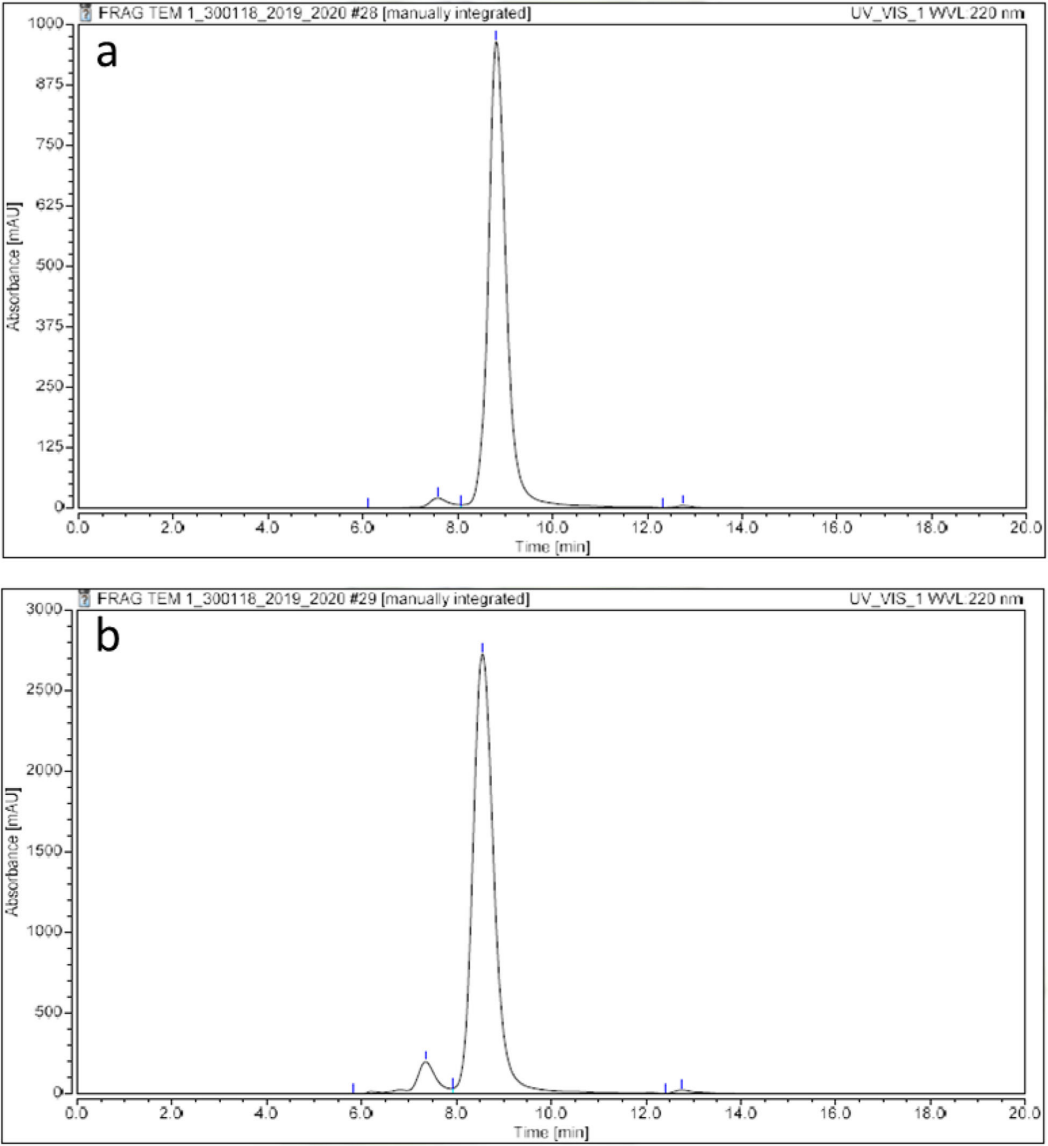
Results of HPLC analysis. **a** Native 1C1m-Fc. **b** 1C1m-Fc conjugated with 20 eq. of DOTA

1C1m-Fc and its conjugates were analyzed by HPLC (Fig. 2).

For native 1C1m-Fc, the main peak was observed at 8.8 min. An aggregate peak at 7.5 min and another peak at 12.7 min were also observed. This profile served as a reference antibody retention time. The area under curve (AUC) of the aggregates increase after conjugation (Table 1).

**Table 1:** HPLC analysis results of the native and conjugated 1C1m-Fc

	Native 1C1m-Fc	1C1m-Fc + 10 equivalents DOTA	1C1m-Fc + 20 equivalents DOTA	1C1m-Fc + 40 equivalents DOTA
% of the main peak	97.4	94.5	92.4	94.9
% of aggregates	2.12	5.3	6.7	4.6
% of other impurities	0.5	0.17	0.75	0.5

Mass spectrometry analysis gave a DOTA conjugation number of two, three, and six respectively for 10, 20, and 40 equivalents of DOTA added (Fig. 2 and Table 1, supplementary data).

**Fig. 3:**
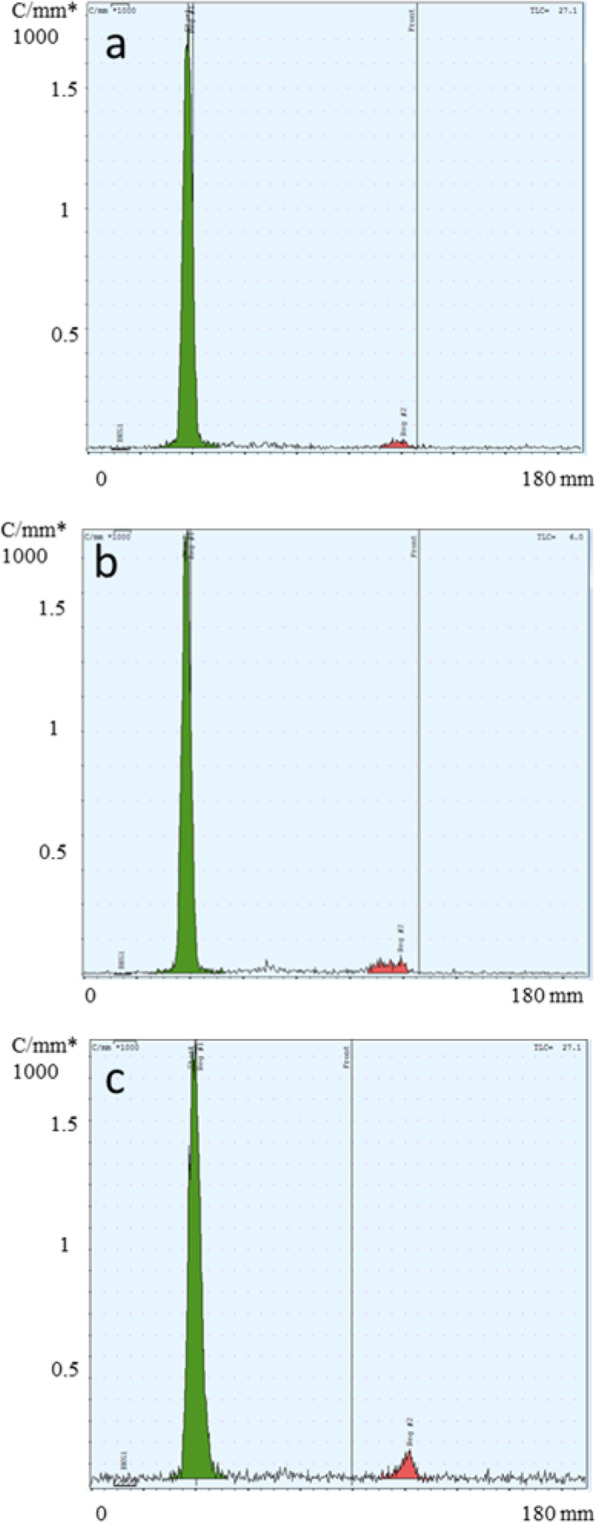
Radio-TLC analysis of [177Lu]Lu-1C1m-Fc (20 MBq of ^177^Lu). **a** After labeling RCP = 97.4%. **b** 24 h after labeling in serum RCP = 95.3%. **c** 48 h after labeling in serum RCP = 93.9%

1C1m-DOTA was successfully radiolabeled with ^177^Lu. The best radiochemical purity, evaluated by radio thin layer chromatography (TLC), was obtained with 20 equivalents of DOTA and the release criteria was 95%. HPLC profile of [177Lu]Lu-1C1m-Fc was assessed by HPLC (Fig. 3, supplementary data). We decided to use this antibody/DOTA ratio for the study. The maximal specific activity was 400 MBq/mg. Stability in serum was also assessed by iTLC and was up to 93% 48 h after labeling (*n* = 1) (Fig. 3).

HPLC showed that the native antibodies (stored at −80 °C) and the conjugates (stored at 2-8 °C) were stable for up to 1 year without any additional formulation.

### Results: In vitro binding

In flow cytometry analysis, native 1C1m-Fc bound to both human (MFI respectively 8959 and 7714 at 2 and 0.2 μg/ml) and murine TEM-1 positive cells.

**Fig. 4:**
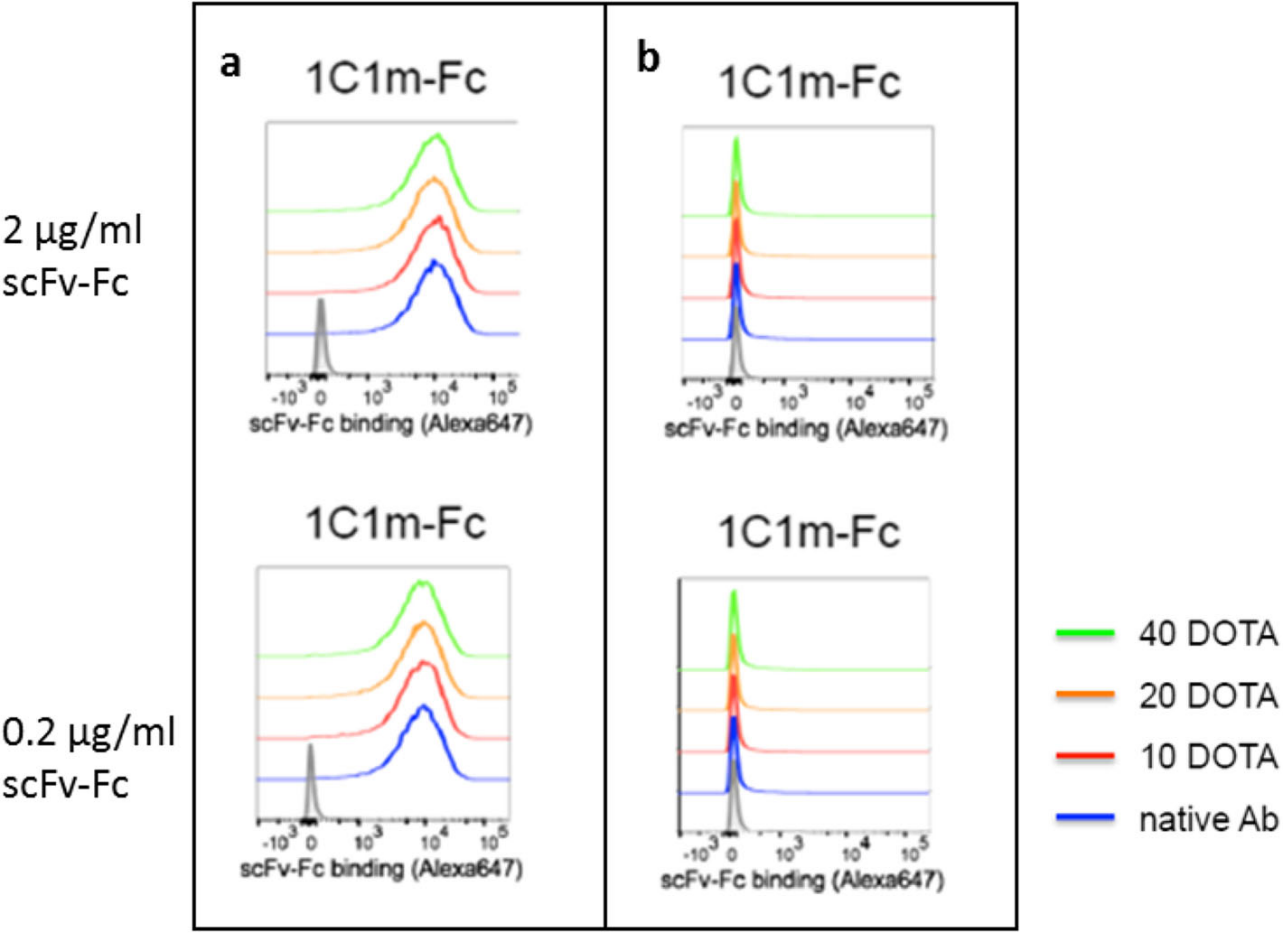
Flow cytometry analysis. **a** Binding to huTEM-1 positive SK-N-AS cells. **b** Binding to huTEM-1 negative HT-1080 cells

For conjugates, the binding to TEM-1 positive cells was respectively 8654, 8095, 8321 at 2 μg/ml for 10, 20, and 40 equivalents of DOTA; 7714, 7679, 7454 at 0.2 μg/ml for 10, 20, and 40 equivalents of DOTA and 58.6 for the isotype control (Fig. 4).

A ratio of 20 DOTA per antibody has been selected for this study to optimize the specific activity of the radiolabeling.

**Fig. 5:**
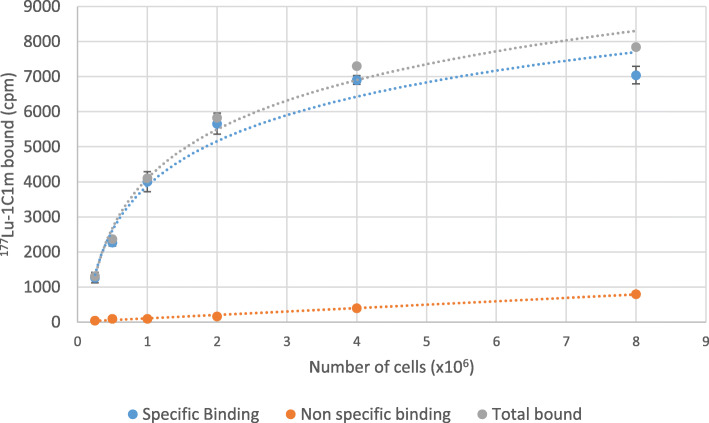
[177Lu]Lu-1C1m-Fc immunoreactivity test on SK-N-AS cell line, binding curve, *B*_max_ = 8490 cpm, total activity = 9460 cpm

The immunoreactivity following the radiolabeling was determined by Lindmo assay and was 86.2% ± 3.9% for [177Lu]Lu-1C1m-Fc (*n* = 2) (Fig. 5).

### Results: In vivo characterization

#### Saturation assay

The biodistribution results of the 1C1m-Fc dose-escalation study are shown in Fig. 4, supplementary data.

The total 1C1m-Fc dose of 50 μg provided the best biodistribution in the tumor and a sufficient specific activity for a theranostic approach. This amount was chosen for the biodistribution experiments.

#### Biodistribution study

**Fig. 6:**
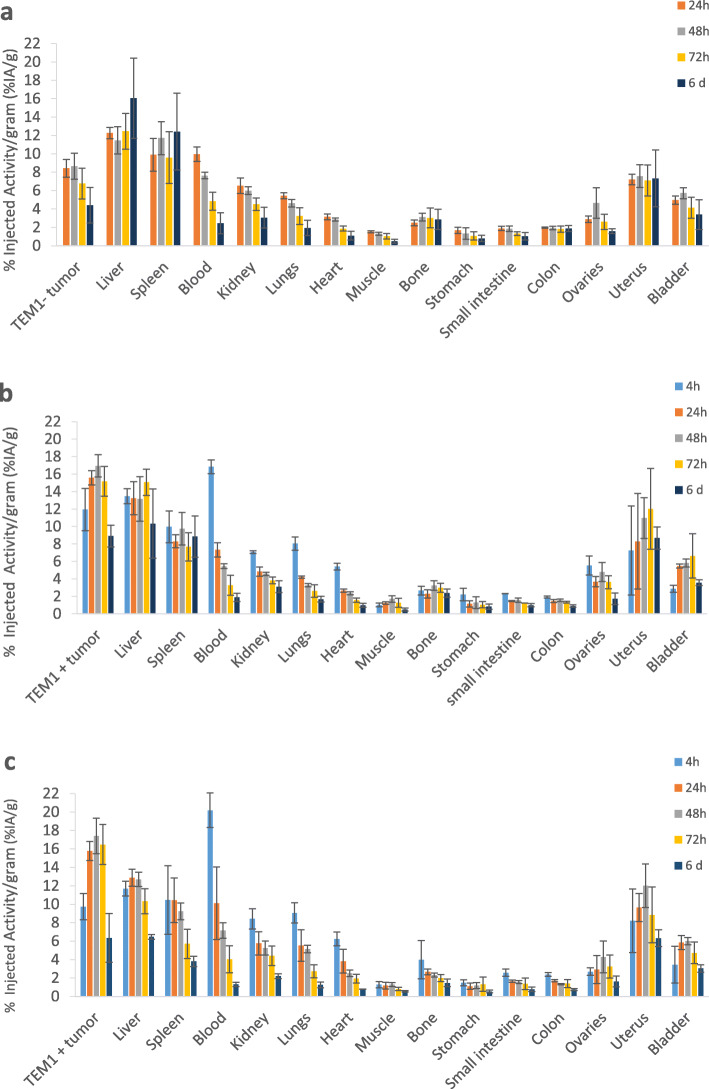
Biodistribution of [177Lu]Lu-1C1m-Fc in Balb/c nu mice. **a** TEM-1 negative tumor (HT-1080)-bearing mice with Kiovig™ preinjection, group 1. **b** TEM-1 positive tumor (SK-N-AS)-bearing mice with Kiovig™ preinjection, group1. **c** TEM-1 positive tumor (SK-N-AS)-bearing mice without Kiovig™ preinjection, group 2. Data are shown as mean ± SD. There were significant differences between uptake in TEM-1 positive tumors compared with TEM-1 negative tumors (*p* = 0.0006 at 24 h). There was no difference on the biodistribution with or without Kiovig™ preinjection (all *p* > 0.059)

A biodistribution study of [177Lu]Lu-1C1m-Fc conjugated with 3 DOTA was performed with and without saturation with Kiovig™ (respectively, groups 1 and 2) (Fig. 6).

For group 1 with Kiovig™ saturation, uptake in TEM-1 negative tumor was significantly lower than TEM-1 positive tumor with uptake clearing over time from 8.4 ± 0.97% IA/g at 24 h (*p* = 0.0006) and 4.4 ± 1.9% IA/g on day 6 (*p* = 0.02). Pre-injection with Kiovig™ had no influence on biodistribution (*p* > 0.05, *t* test). For group 1 and 2, uptake in TEM-1 positive tumor was 10.8% ± 1.55 IA/g 4 h after injection and remained consistently high even 3 days after injection (15.8% ± 1.9 IA/g), demonstrating retention of TEM-1 targeted antibody. Uptake in TEM-1 positive tumor decreased to 7.6 ± 1.8% IA/g 6 days after injection.

A second experiment was done with the same conditions as for group 2, without Kiovig™ saturation, but with [177Lu]Lu-1C1m-Fc conjugated with 6 DOTA. The uptake in the liver and in the spleen was 79% ± 12.5 IA/g and 82% ± 40 IA/g, respectively, at 24 h (Table 2).

**Table 2:** Liver and tumor uptake regarding the estimated number of DOTA fixed on 1C1m-Fc

Estimated number of DOTA	Liver uptake (%IA/g) (*T* = 24 h)	Tumor TEM-1 positive uptake (%IA/g) (*T* = 24 h)
6	79 ± 12	11.6 ± 0.3
3	12.8 ± 0.9	15.8 ± 1

#### SPECT/CT study

**Fig. 7:**
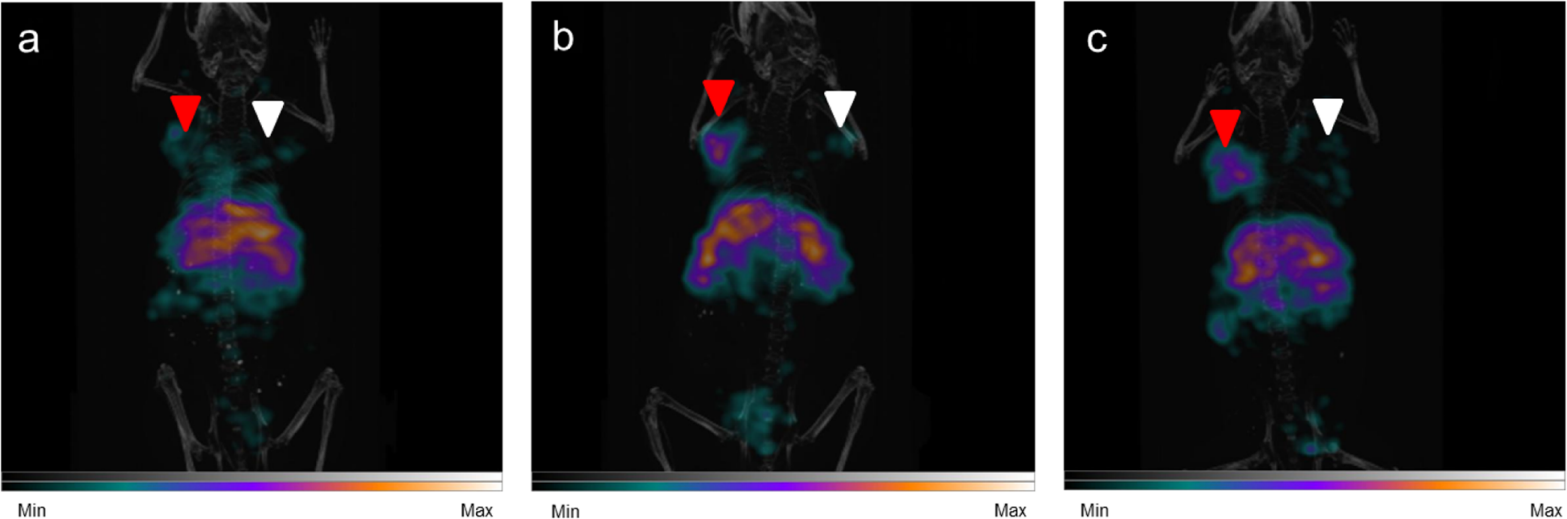
[177Lu]Lu-1C1m-Fc dorsal view SPECT/CT fusion maximum intensity projection imaging on mice with TEM-1 positive tumor (SK-N-AS, left flank, red arrow) and TEM-1 negative tumor (HT-1080, right flank, white arrow), (**a**) at 24 h, (**b**) at 48 h, (**c**) at 72 h

The SPECT/CT imaging showed a specific uptake in TEM-1 positive tumor (Fig. 7) and liver uptake. The uptake ratio between TEM-1 positive tumor and TEM-1 negative tumor was determined at 24, 48, and 72 h (Table 3). A 1.9-fold higher signal at 72 h was observed in SPECT/CT imaging in TEM-1 positive tumors versus control tumors.

**Table 3:** SPECT imaging of tumor uptake at 24, 48, 72 h

Time post-injection	SK-N-AS (Cps/tumor volume)	HT-1080 (Cps/tumor volume)	Ratio SK-N-AS/HT-1080
24 h	2.17E+07	1.61E+07	1.3
48 h	3.17E+07	1.85E+07	1.7
72 h	3.03E+07	1.60E+07	1.9

The signal ratio TEM-1 positive to TEM-1 negative tumor obtained with the SPECT/CT at 72 h was similar to the one obtained in biodistribution (with a factor of 2.2 and 1.9, respectively).

#### Dosimetry

Extrapolated organ absorbed doses for mice derived from the injection of [177Lu]Lu-1C1m-Fc are reported in Table 4.

**Table 4:** Mouse dosimetry of [177Lu]Lu-1C1m-Fc. These estimates come from mouse TIACs calculated from source organ time-activity curves

Source organ	Mouse average organ mass (g)	Average TIAC (MBq·h/MBq)	Absorbed dose (mGy/MBq)
Tumor SK-N-AS	0.16	3.91	1.82E+03
Liver	1.28	30.42	2.23E+03
Kidneys	0.33	2.04	7.05E+02
Lung	0.15	0.71	5.39E+02
Spleen	0.09	1.12	1.20E+03
Heart	0.20	0.33	3.63E+02
Stomach	0.10	0.55	1.15E+03
Small intestine	1.38	2.73	4.38E+02
Colon	0.87	1.74	3.28E+02
Bladder	0.10	0.13	3.16E+02
Uterus*	0.08	1.66	1.5 E+03
Total body	20.19	98.78	4.25E+02

*The uterus is not part of the source/target organ in the murine model of the OLINDA/EXM 2.1 software. Specific dosimetry was obtained with the sphere model of the OLINDA/EXM 2.1 where the organ-specific average mass and TIAC were applied

The organ receiving the highest absorbed dose was determined to be the liver (2.23 Gy/MBq), followed by the uterus (1.5 Gy/MBq), the spleen (1.2 Gy/MBq) and the stomach (1.15 Gy/MBq). The total body dose was 0.4 Gy/MBq and the tumor dose was 1.82 Gy/MBq.

## Discussion

Two major challenges in the field of theranostics must be considered: firstly, the identification of suitable tumor-specific targets and secondly, the development of high-affinity antibodies. Ideal targets should present the following criteria: high and exclusive expression in tumors and a broad expression across a variety of tumor types, affording opportunities for universal cancer therapies.

TEM-1 is a robust target overexpressed specifically in the tumor vasculature of a large number of adenocarcinomas. Tumor vasculature cells provide critical support for tumor survival, growth, and invasion and act as physical and molecular barriers that protect tumor cells from the host immune system [27, 28]. In addition, endothelial cells are accessible directly via the bloodstream.

Sarcoma is a heterogeneous group of tumors with a high unmet medical need. The literature consistently reports a strong expression of TEM-1 in sarcomas, which is localized in malignant cells and perivascular and stromal cells, allowing simultaneous targeting of tumor cells and the tumor vasculature [17–19].

Given the very short half-life and the relative in vivo instability of monovalent scFv antibody fragments, a bivalent Fc-fusion protein based on a novel single-chain antibody, 1C1m-Fc, was chosen for evaluation in this study. The fusion of scFvs to the IgG Fc constant domains adds significant size, avidity, and stability to the targeting moiety and would be expected to lead to improved blood pharmacokinetics. In contrast to the previously described anti-TEM-1 MORAb-004 antibody, the cross-reactivity of 1C1m toward both human and murine TEM-1 allows the evaluation of anti-TEM-1 theranostic approaches in the mouse.

1C1m-Fc was conjugated and radiolabeled with ^177^Lu to allow its use both for SPECT imaging and for the potential delivery of a therapeutic payload. We have therefore performed a preliminary preclinical evaluation of this fusion protein. The conjugation and the radiolabeling process were optimized to obtain a radiochemical purity up to 95%.

For the in vitro characterization, 1C1m-Fc and its conjugates were tested for binding to TEM-1 using flow cytometry analysis. A high percentage of binding was observed for 1C1m-Fc at each concentration tested and for each ratio used in the coupling reaction. A concentration of 20 equivalents of DOTA has been chosen as suitable for achieving an optimal specific activity of radiolabeling.

The results of the Lindmo analysis demonstrated that the radiolabeling did not affect the immunoreactivity.

A first biodistribution study using [177Lu]Lu-1C1m-Fc with co-injection of Kiovig™ was performed. The aim was to examine the influence of Fc receptor blocking on biodistribution and uptake, as mouse Fc receptors are highly abundant in mouse spleen and liver and are known to show appreciable binding to human Fc [29]. Kiovig™ is a readily available commercial IgG that does not bind or mask TEM-1 on SK-N-AS cells.

The results of this biodistribution experiment were compared with a second study arm performed without Kiovig™ co-injection. This experiment revealed specific tumor targeting in the two mouse xenograft models with no impact of the Kiovig™ co-injection on biodistribution. The [177Lu]Lu-1C1m-Fc was found to enrich mainly in the liver and the spleen, as has been observed for many antibodies [28, 30, 31]. Quantitative PCR, performed by other groups on biopsies taken from mice upon sacrifice has shown an absence of detectable TEM-1 in the liver, confirming this uptake to be non-specific in nature.

As no difference was shown with or without saturation, we asked whether the liver and the spleen uptake could be explained by the number of conjugated DOTA moieties on the fusion protein. Indeed, the number of DOTA attached per antibody can vary depending on the molar ratios of both the antibody and DOTA used for the conjugation. To achieve a high radiolabeling efficiency and probe sensitivity it is often desirable to conjugate a higher number of chelators per antibody. However, the hydrophilic character of DOTA can significantly perturb the lipophilicity/hydrophilicity properties of the acceptor antibody with unpredictable consequences for pharmacokinetics.

Regarding the DOTA conjugate, antibody ratio, other authors have reported that a reduction of non-specific hepatic uptake is correlated with an increased number of DOTA per antibody. Indeed, it has been suggested that the negative charge conferred to the antibody by DOTA conjugation results in a reduced isoelectric point (pI), causing a net repulsion between the molecule and the phospholipid bi-layer [32, 33]. However, Rinne et al. working with gallium-68 and indium-111 did not observe a clear relationship between the extent of negative charge and uptake [32]. As isotopes differ in charge, coordination number, and chelation geometry, the biodistribution of any conjugate is likely to be significantly influenced by not only the choice of targeting antibody but also by the combination of chelator and radio-isotope, in addition to the conjugation ratio. Hence, each conjugate should be optimized accordingly [32, 34].

In our study, the 1C1m-Fc fusion protein was conjugated with DOTA and radiolabeled with ^177^Lu. The number of DOTA per antibody was evaluated by mass spectrometry and the uptake and retention in the liver was found to increase with the number of DOTA fixed on 1C1m-Fc. Our results are in accordance with reports showing that a high number of chelators coupled to an antibody can result in accelerated blood clearance and high liver uptake [34, 35]. Moreover, at high DOTA conjugation ratios, the possibility of DOTA attachment to important residues in the antigen-targeting variable domains of the antibody increases, potentially compromising the immunoreactivity of the molecule [36]. Additionally, high chelator conjugation ratios could cause conformational changes to an antibody that can result in rapid sequestration of the radio conjugate into the liver and spleen, as well as accelerated uptake by the reticuloendothelial system, resulting in unfavorable pharmacokinetic properties [37]. Elevated uptake in the liver can also indicate a lower stability of the radiolabeling such as trans-chelation to transferrin [38].

We observed that an optimization of the tumor/liver ratio could be achieved by reducing the number of DOTA coupled to 1C1m-Fc. We have performed a conventional conjugation, which can produce heterogeneous mixtures with respect to conjugate ratio and sites of conjugation. In this case, site-specific conjugation could be a technique to be evaluated for the improvement of batch to batch consistency of the conjugates and to avoid the potential risks of non-specific conjugation [39, 40].

Although the binding of 1C1m has been shown to be TEM-1 specific, we have observed significant retention of the conjugates in TEM-1 negative tumors. This uptake could be explained by possible TEM-1 expression by the neo-vessels that form in these tumors. In this case, the antibodies are probably distributed only in the neo-vessels and not retained by any binding to tumor cells. As the appearance of the tumors was highly vascularized, this stasis in the neovessels could be due to the EPR effect in the neovessels. These hypotheses will be tested in future experiments using fluorescence microscopy.

Biodistribution data were used for dosimetry calculations. The organ receiving the highest absorbed dose would be the liver (2.23 Gy/MBq) followed by the uterus (1.5 Gy/MBq), the spleen (1.2 Gy/MBq), and the stomach (1.15 Gy/MBq). The total body dose would be (0.4 Gy/MBq) and the tumor dose 1.82 Gy/MBq. In particular, a specific dose estimate was performed for the uterus using the spherical model available in the OLINDA/EXM 2.1 software. This approach does not consider the specific morphology of the organ but it is at present the best approximation we can provide without applying specific Monte Carlo dose calculations which are beyond the scope of this study.

To evaluate the organ radiotoxicity after therapy administration in humans it would be necessary to extrapolate the human dosimetry to from the pre-clinical model. This study will be the subject of a forthcoming analysis in which the extrapolation will be performed for the optimized radiolabeled compound.

We have performed SPECT/CT imaging experiments on an scFv-Fc antibody that binds both human and mouse TEM-1, and generated data showing that TEM-1 was either absent or present at negligible levels in normal mouse organs and that [177Lu]Lu-1C1m-Fc was able to efficiently target a TEM-1 positive tumor in vivo. The imaging at the 72 h timepoint compared with the biodistribution data for the same mouse showed that the results of the two techniques can be complementary.

Hence, SPECT molecular imaging of sarcoma or other solid tumors could contribute in radiologic staging and in applications such as the preoperative evaluation of patients to assess tumor resectability or radio-guided surgery of metastases.

## Conclusion

The highly specific expression of TEM-1 in several types of solids tumors suggests that [177Lu]Lu-1C1m-Fc could prove a potentially useful and safe tool for molecular imaging and theranostic applications.

The number of DOTA molecules attached per antibody moiety plays a significant role in determining the success of tumor targeting employing radiolabeled antibodies [36]. Further experiments could be done to find the best ratio of DOTA per antibody to maintain a balance between radiochemical yield, immunoreactivity, and pharmacokinetic behavior to develop an optimal radiolabeled 1C1m-Fc suitable for theranostic application.

## Supplementary information

**Additional file 1: **Supplementary data. **Figure S1**. SDS-page in non-reducing (**b, c, d**) and reducing conditions (**e, f, g**) using NuPAGE Bis-Tris gradient gels. (**a)** marker; (**b, e**) native 1C1m-Fc; (**c, f**) 1C1m-Fc conjugated with 3 DOTA; **(d, g)** 1C1m-Fc conjugated with 6 DOTA. **Figure S2**. Mass spectrometry analysis of native 1C1m-Fc (**a**) and of 1C1m-Fc conjugated with 3 DOTA (**b**). **Figure S3**. HPLC profile of [177Lu]Lu-1C1m-Fc conjugated with 3 DOTA. **Figure S4**. Saturation assay in Balb/c nu mice. 2.5 μg of [177Lu]Lu-1C1m-Fc conjugated with 3 DOTA was co-injected with an increasing amount of unlabeled native 1C1m-Fc (respectively 2.5, 50, 100, 200 and 500 μg). The %IA/g was evaluated at 24 hours. **Table S1**. Estimated number of DOTA per 1C1m-Fc based on mass spectrometry analysis

## Data Availability

The datasets used and/or analyzed during the current study are available from the corresponding author on reasonable request.
