# Supplementary material for: 177Lu radiolabeling and preclinical theranostic study of 1C1m-Fc: an anti-TEM-1 scFv-Fc fusion protein in soft tissue sarcoma
Source: EJNMMI Res. 2020 Aug 17;10:98. doi: 10.1186/s13550-020-00685-3 (PMC7431510; doi:10.1186/s13550-020-00685-3)
Supplement: Supplementary file 1 — Additional file 1: Supplementary data. Figure S1. SDS-page in non-reducing (b, c, d) and reducing conditions (e, f, g) using NuPAGE Bis-Tris gradient gels. (a) marker; (b, e) native 1C1m-Fc; (c, f) 1C1m-Fc conjugated with 3 DOTA; (d, g) 1C1m-Fc conjugated with 6 DOTA. Figure S2. Mass spectrometry analysis of native 1C1m-Fc (a) and of 1C1m-Fc conjugated with 3 DOTA (b). Figure S3. HPLC profile of [177Lu]Lu-1C1m-Fc conjugated with 3 DOTA. Figure S4. Saturation assay in Balb/c nu mice. 2.5 μg of [177Lu]Lu-1C1m-Fc conjugated with 3 DOTA was co-injected with an increasing amount of unlabeled native 1C1m-Fc (respectively 2.5, 50, 100, 200 and 500 μg). The %IA/g was evaluated at 24 hours. Table S1. Estimated number of DOTA per 1C1m-Fc based on mass spectrometry analysis [file 13550_2020_685_MOESM1_ESM.docx]

**SUPPLEMENTARY DATA**

**Figure 1.** SDS-page in non-reducing (**b, c, d**) and reducing conditions (**e, f, g**) using NuPAGE Bis-Tris gradient gels. (**a)** marker; (**b, e**) native 1C1m-Fc; (**c, f**) 1C1m-Fc conjugated with 3 DOTA; **(d, g)** 1C1m-Fc conjugated with 6 DOTA.


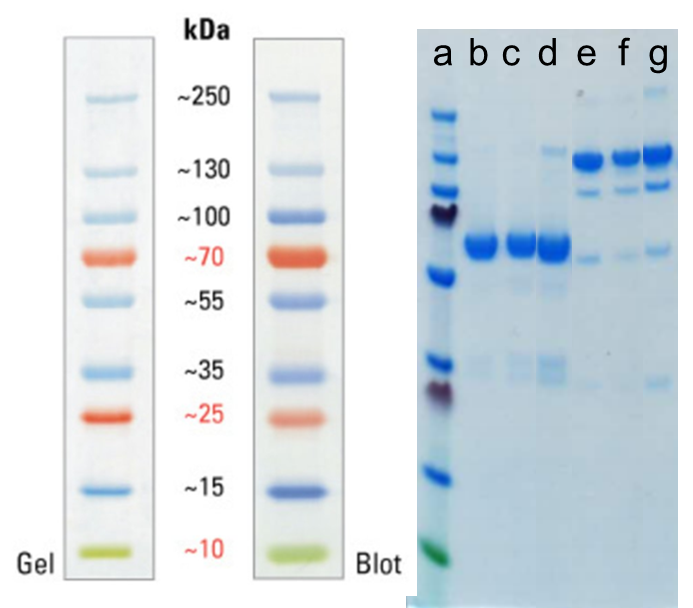


**Figure 2.** Mass spectrometry analysis of native 1C1m-Fc (**a**) and of 1C1m-Fc conjugated with 3 DOTA (**b**)

**
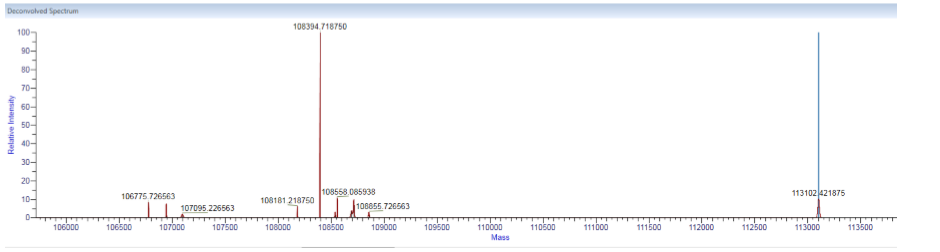
**

**a**

**
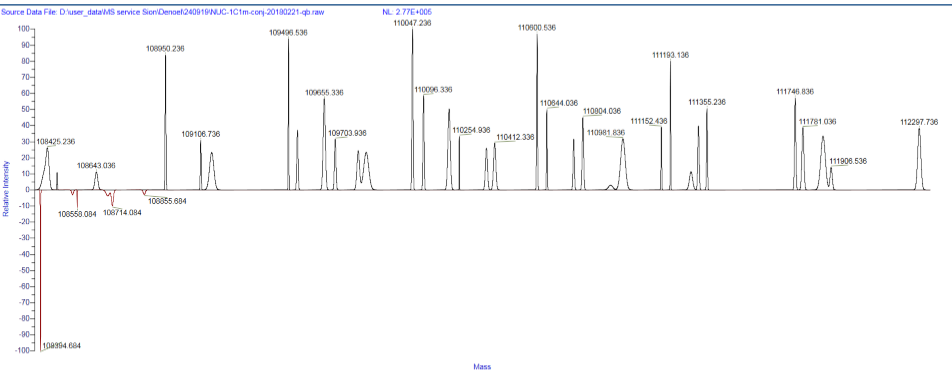
**

**b**

**Figure 3.** HPLC profile of [177Lu]Lu-1C1m-Fc conjugated with 3 DOTA


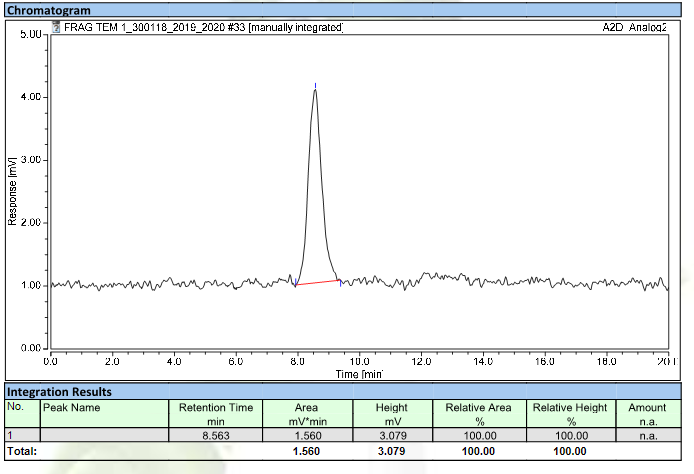


**Figure 4.** Saturation assay in Balb/c nu mice. 2.5 µg of [177Lu]Lu-1C1m-Fc conjugated with 3 DOTA was co-injected with an increasing amount of unlabeled native 1C1m-Fc (respectively 2.5, 50, 100, 200 and 500 μg). The %IA/g was evaluated at 24 hours.

**Table 1.** Estimated number of DOTA per 1C1m-Fc based on mass spectrometry analysis

|  | **Mass Weight (Da)** | **Estimated number of DOTA per 1C1m-Fc** | **Average number of DOTA per 1C1m-Fc** |
| --- | --- | --- | --- |
| **1C1m-Fc native** | 108394 | NA | NA |
| **DOTA (- HCl - H2O)** | 551 | NA | NA |
| **1C1m-Fc 10 eq DOTA** | 108986 to 110758 | 1-3 | 2 |
| **1C1m-Fc 20 eq DOTA** | 109496 to 111746 | 2-4 | 3 |
| **1C1m-Fc 40 eq DOTA** | 110715 to 112487 | 4-8 | 6 |
